# Case Report: Spontaneous regression of cystic renal cell carcinoma

**DOI:** 10.3389/fonc.2026.1755717

**Published:** 2026-06-17

**Authors:** Zuhuan Xu, Yunfeng Zhang, Penglin Zhang, Honglin Hu

**Affiliations:** Department of Urology, The Second Affiliated Hospital, Jiangxi Medical College, Nanchang University, Nanchang, China

**Keywords:** Bosniak grade, case report, clear cell renal cell carcinoma, cystic renal cell carcinoma, spontaneous regression, tumor microenvironment

## Abstract

**Background:**

Spontaneous regression (SR) of cystic renal cell carcinoma (CRCC) is extremely rare, and previous reports lack pathological confirmation. In this review, we report a case of spontaneous partial regression of Bosniak grade IV CRCC confirmed by postoperative pathology, and discuss its potential mechanism.

**Case summary:**

A 59-year-old female patient with a simple cyst (34*31 mm) in her left kidney was detected by color doppler flow imaging(CDFI) in July 2023. In October 2024, the computed tomography(CT) showed that the lesion had progressed to Bosniak grade IV cystic mass (60*50 mm), which was not treated. Follow-up magnetic resonance imaging (MRI) in April 2025 showed that the lesion was significantly reduced (25*28 mm). The patient underwent robot-assisted laparoscopic partial nephrectomy, and was pathologically diagnosed with clear cell renal cell carcinoma(ccRCC) after surgery.

**Conclusions:**

Despite spontaneous regression, Bosniak grade IV renal cystic disease requires surgical intervention to confirm the diagnosis and avoid progression. This study provides a new basis for the natural history of high-risk renal cystic lesions and individualized follow-up strategies.

## Background

Spontaneous regression (SR) of cancer is a relatively rare phenomenon in clinical practice (1). SR refers to the phenomenon that the volume of malignant tumors partially or completely shrinks or even disappears after no targeted medical treatment (such as surgery, chemotherapy, radiotherapy, etc.), or only after receiving treatments that are considered ineffective (1, 2). Renal cell carcinoma (RCC) is one of the most common malignancies reported in the literature, and most of them are solid RCCs (3). Here, we report a case of pathologically confirmed spontaneous partial regression of cystic renal cell carcinoma(CRCC) and explore the possible mechanism of this phenomenon.

## Case report

The patient, a 59-year-old female of Han Chinese ethnicity, had a history of hypertension without other significant medical conditions and presented for follow-up evaluation of a renal cyst. On July 27, 2023, during a routine physical examination that included a color Doppler flow imaging (CDFI) study, the patient was initially found to have a simple renal cyst (Bosniak category I) measuring approximately 34*31 mm in the left kidney (**Figure 1**), for which observational follow-up was recommended. On October 31, 2024, the patient returned to the outpatient clinic for follow-up examination, and the computed tomography (CT) examination showed that the left kidney had a cystic lesion (Bosniak grade IV), about 60*50 mm in size (**Figure 2**). From the initial detection in July 2023 until the follow-up examination in October 2024, the patient did not seek medical consultation or undergo any additional examinations. Following this outpatient follow-up examination, the patient was not hospitalized for further diagnosis and treatment, as she had not checked the CT report in a timely manner and we were unable to contact her. Finally, on April 9, 2025, the patient returned to the outpatient clinic. Due to the aforementioned reasons, she was unaware of her actual medical condition, which led to her returning for follow-up only after half a year. After reviewing her historical reports, we informed her of her condition and recommended hospitalization for further evaluation and treatment. During this visit, the patient underwent magnetic resonance imaging (MRI), which revealed significant shrinkage of the left renal cystic lesion (Bosniak grade IV) to approximately 25*28 mm (**Figure 3**). During this time, the patient did not receive any treatment, had no history of trauma, and showed no signs of cyst rupture on imaging. All laboratory findings on admission, including complete blood count, liver and kidney function tests, electrolyte panel, coagulation profile, and urinalysis, were within normal limits. Lactate dehydrogenase was also normal. However, the erythrocyte sedimentation rate (ESR) was elevated at 38 mm/h (reference range: 0–20 mm/h).

**Figure 1:**
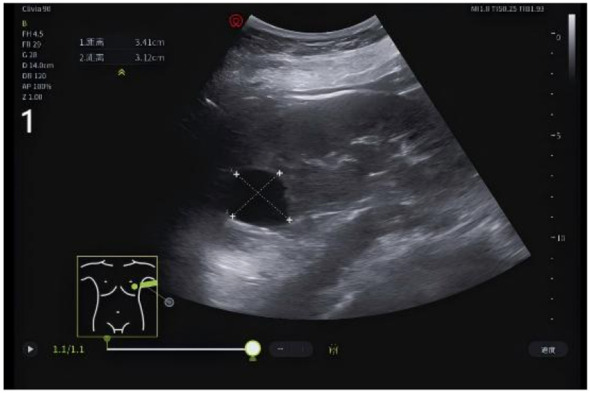
The patient's color doppler flow imaging (CDFI) on July 27, 2023 showed a cystic lesion of about 34*31 mm in size in the left renal parenchyma, with thin walls, clear borders, and no obvious blood flow signal inside, which was a simple renal cyst (Bosniak grade I).

**Figure 2:**
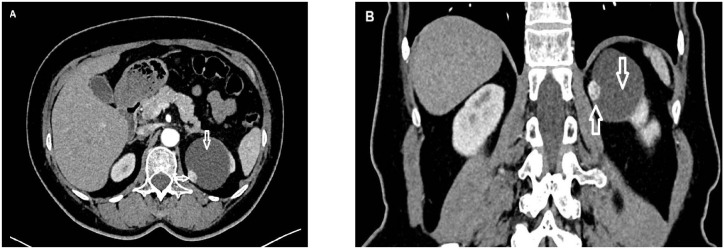
**(A)** Axial CT image. **(B)** Coronal CT image. The patient's computed tomography (CT) scan on October 31, 2024 showed a cystic mass at the upper pole of the left kidney, about 60×50 mm in size, and a markedly enhancing soft tissue nodule on the inner edge, Bosniak grade IV.

**Figure 3:**
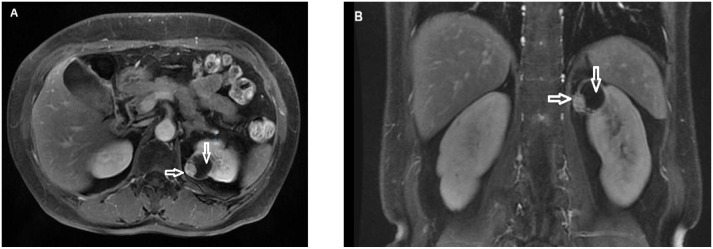
The magnetic resonance imaging (MRI) on April 9, 2025 showed a cystic mass at the upper pole of the left kidney, about 25*28 mm in size, with a thick cyst wall and significantly strengthened adherent nodule, Bosniak grade IV. The size of the lesion is significantly smaller than before (The most significant volume reduction was due to the reduction of cystic fluid volume. No significant reduction was observed in the solid component).

Although follow-up imaging over six months demonstrated considerable shrinkage of the lesion, definitive surgical intervention with robotic-assisted laparoscopic partial nephrectomy was pursued, given the well-established malignant risk of Bosniak grade IV renal cystic lesions. Postoperative histopathological examination showed clear cell renal cell carcinoma(ccRCC) (**Figure 4**) and no cancer involvement was found at the resection margin. After the surgery, we cut a specimen of a postoperatively removed mass, in which the fluid was brown, viscous, jelly-like, and contained bloody material (**Figure 5**). The patient recovered well after surgery and was discharged from the hospital smoothly, and is continuing to follow up (**Figure 6**).

**Figure 4:**
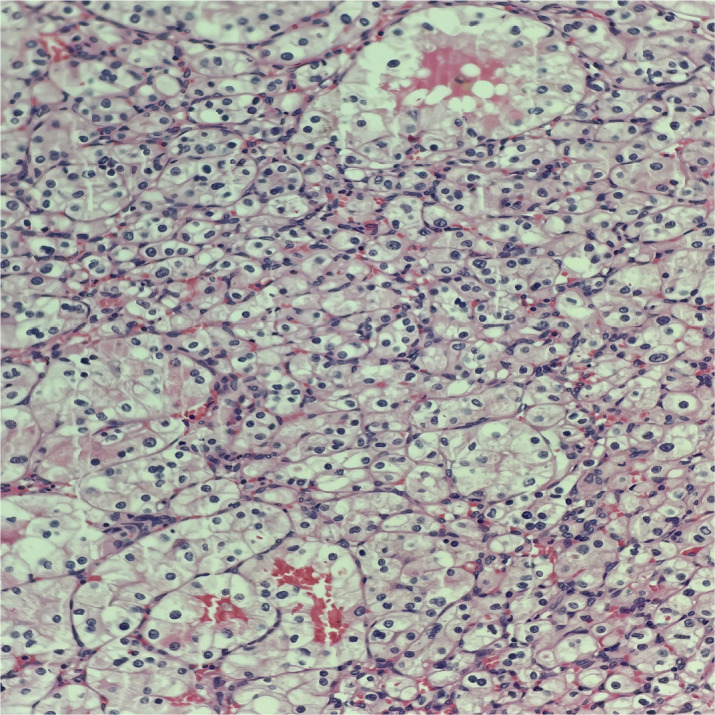
**(A)** Axial MRI image. **(B)** Coronal MRI image. The magnetic resonance imaging (MRI) on April 9, 2025 showed a cystic mass at the upper pole of the left kidney, about 25×28 mm in size, with a thick cyst wall and a markedly enhancing adherent nodule, Bosniak grade IV. The lesion was significantly smaller than before, mainly due to the reduction in cystic fluid volume, whereas no significant reduction was observed in the solid component.

**Figure 5:**
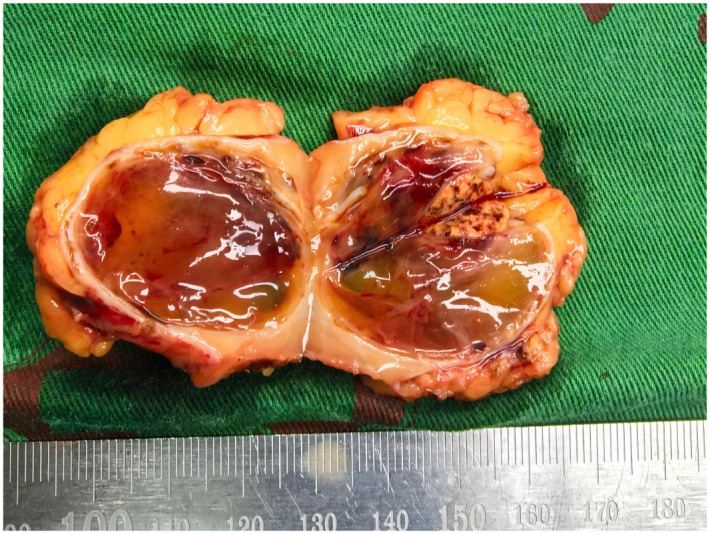
An incision of the lump reveals a solid nodule with brown, viscous, jelly-like fluid containing bloody material.

**Figure 6:**
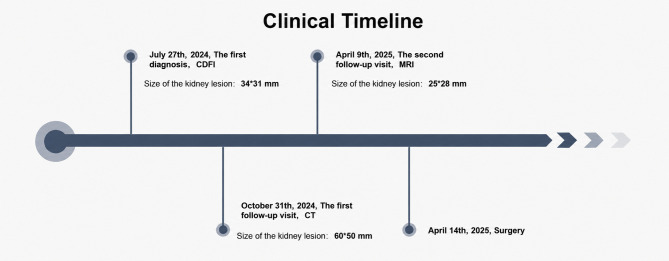
A clinical timeline: It records the timeline of the patient from the first diagnosis until the time of the surgery.

## Discussion

The Bosniak classification is a classification system for grading cystic lesions of the kidney based on imaging examinations, which is mainly used to assess the benign and malignant risk of kidney cysts, so as to provide guidance for clinical diagnosis and treatment. Within the Bosniak classification system, category III and IV cystic renal masses harbor a significant risk of malignancy (4). Bosniak IV lesions are considered surgical diseases mandating intervention, while most Bosniak III lesions also warrant strong consideration for resection, typically following multidisciplinary evaluatio (4). While SR of Bosniak III/IV lesions had been documented, the definitive pathological nature of these regressing lesions remained speculative due to the absence of surgical confirmation (5, 6). Our case provided histopathological proof of malignancy in a regressing Bosniak IV cyst.

Due to the rarity and particularity of SR of cancer, the mechanism has always been the focus of attention and discussion among scholars.

Immune-mediated responses and alterations in the tumor microenvironment (TME) are key mechanisms in RCC spontaneous regression (7, 8). The TME refers to the surrounding environment in which tumor cells are located, including extracellular matrix, blood vessels, immune cells, etc. It can directly provide nutrients and growth factors that promote tumor cell proliferation and metastasis, and plays an important role in cancer growth and progression (9). In RCC, the TME may also induce the occurrence of related immune responses through the complex interaction of various immune cells, cytokines, and signaling pathways, directly affecting the growth and proliferation of tumor cell (10).

In this case, imaging studies (CT and MRI) revealed significant lesion reduction, primarily characterized by a decrease in the cystic component, likely due to the reabsorption of cystic fluid. Concurrently, the elevated ESR may be associated with an immune response (11). Upon incision, the mass was found to contain viscous, blood-tinged fluid, possibly resulting from intracyptic hemorrhage or ischemic necrosis. A cascade of immune responses induced by changes in the TME secondary to intracapsular avascular necrosis or hemorrhage may be the key mechanism of its shrinkage.

However, our study also has its limitations. Being a single case report, the proposed mechanisms for SR described above are reasonable inferences based on available evidence, but they currently lack direct immunological or molecular proof. we lack serial fluid or tissue samples to dynamically analyze cytokine profiles or immune cell infiltration within the cyst. Future studies encountering similar cases could employ cyst fluid aspiration prior to resection for biomarker analysis. Furthermore, dedicated volumetric segmentation of the solid component on imaging would clarify whether regression was truly isolated to the cystic area.

## Conclusion

We report a pathologically confirmed case of SR of CRCC and discuss its potential underlying mechanisms. Despite evidence of SR, surgical resection remains the standard of care for Bosniak IV renal cystic lesions due to their high malignant potential. This case provides an important basis for exploring the natural history of cystic kidney cancer and individualized follow-up strategies.

## Data Availability

The original contributions presented in the study are included in the article/Supplementary Material. Further inquiries can be directed to the corresponding author.
